# Training deep learning based dynamic MR image reconstruction using open-source natural videos

**DOI:** 10.1038/s41598-024-62294-7

**Published:** 2024-05-23

**Authors:** Olivier Jaubert, Michele Pascale, Javier Montalt-Tordera, Julius Akesson, Ruta Virsinskaite, Daniel Knight, Simon Arridge, Jennifer Steeden, Vivek Muthurangu

**Affiliations:** 1 UCL Centre for Translational Cardiovascular Imaging, University College London, 30 Guilford St, London, WC1N 1EH UK; 2 Clinical Physiology, Department of Clinical Sciences Lund, Lund University, Lund, Sweden; 3 Department of Cardiology, Royal Free London NHS Foundation Trust, London, NW3 2QG UK; 4 Department of Computer Science, University College London, London, WC1E 6BT UK

**Keywords:** Real-time, Dynamic MRI, Deep learning, Image reconstruction, Machine learning, Computer science, Magnetic resonance imaging

## Abstract

To develop and assess a deep learning (DL) pipeline to learn dynamic MR image reconstruction from publicly available natural videos (Inter4K). Learning was performed for a range of DL architectures (VarNet, 3D UNet, FastDVDNet) and corresponding sampling patterns (Cartesian, radial, spiral) either from true multi-coil cardiac MR data (N = 692) or from synthetic MR data simulated from Inter4K natural videos (N = 588). Real-time undersampled dynamic MR images were reconstructed using DL networks trained with cardiac data and natural videos, and compressed sensing (CS). Differences were assessed in simulations (N = 104 datasets) in terms of MSE, PSNR, and SSIM and prospectively for cardiac cine (short axis, four chambers, N = 20) and speech cine (N = 10) data in terms of subjective image quality ranking, SNR and Edge sharpness. Friedman Chi Square tests with post-hoc Nemenyi analysis were performed to assess statistical significance. In simulated data, DL networks trained with cardiac data outperformed DL networks trained with natural videos, both of which outperformed CS (*p* < 0.05). However, in prospective experiments DL reconstructions using both training datasets were ranked similarly (and higher than CS) and presented no statistical differences in SNR and Edge Sharpness for most conditions.The developed pipeline enabled learning dynamic MR reconstruction from natural videos preserving DL reconstruction advantages such as high quality fast and ultra-fast reconstructions while overcoming some limitations (data scarcity or sharing). The natural video dataset, code and pre-trained networks are made readily available on github.

## Introduction

Real-time magnetic resonance (MR) imaging allows evaluation of dynamic changes without relying on physiological gating. Real-time MR is used to examine the heart (particularly in children, during exercise or for interventional applications), assess joint motion, evaluate speech and measure bowel motility^1^. However, real‐time imaging often requires significant data undersampling to ensure adequate spatio-temporal resolution. Therefore, it is usually combined with advanced reconstruction techniques to produce artifact‐free images, with the previous state-of-the-art being Compressed Sensing (CS)^2^.

Unfortunately, there are drawbacks to CS including computationally intensive, time‐consuming reconstructions and unnatural looking images. Recently, it has been shown that supervised Deep Learning (DL) can outperform CS in terms of reconstruction time and image quality^3^. For instance, DL networks applied as a low latency, single-pass, post-processing step (deep artifact suppression) can successfully remove undersampling artifacts from radial^4^ and spiral images^5^. These approaches are potentially useful for applications that require very short inference times such as interventional and exercise MR. However, deep artifact suppression is less successful for Cartesian undersampling^6^, and often requires large amounts of image-based training data^4^. An alternative approach is to use unrolled DL architectures (e.g. VarNet) that have been shown to outperform CS and deep artifact suppression for undersampled Cartesian acquisitions^7^. Although these methods benefit from the inclusion of data consistency, they are slower than deep artifact suppression and importantly require k-space training data.

Currently, one of the main impediments to the development of DL reconstructions is the need for application-specific image or k-space training data. This is particularly true for dynamic MR applications where it can be difficult to obtain large amounts of training data for some applications (e.g. speech imaging). Furthermore, even when application specific training data can be acquired, it is often difficult to share due to data governance issues. We believe the lack of accessible training data is a significant barrier to developing both deep artifact suppression and iterative DL-based MR reconstructions and we propose an alternative approach that leverages readily available natural videos to create dynamic data for training. It has previously been shown that static 2D natural images (e.g. photographs of animals or scenery) can be used to pre-train DL networks for reconstruction of static 2D MR images^8^. We build on this work, using natural videos (e.g. moving cars, animals, and people) to train 2D + time DL models that can reconstruct undersampled dynamic real-time MR data. We used a large open-source database of high-quality natural videos -Inter4K^9^- and the aims of this study were: (1) to demonstrate that it was possible to create synthetic multi-coil complex k-space data for natural videos, (2) use this synthetic data to train three reconstructions using different sampling patterns and state-of-the-art DL based models, and (3) evaluate reconstruction quality on prospectively acquired real-time cardiac cine and speech cine data compared with DL models trained using dynamic cardiac MR data and CS reconstructions.

## Materials and methods

This study conformed to the principles of the Declaration of Helsinki and was approved by the UK National Health Service, Health Research Authority, Research Ethics Committees and written informed consent was obtained in prospective subjects and retrospective data (ref. 21/EE/0037, 17/LO/1499).

### Experimental overview

**Figure 1:**
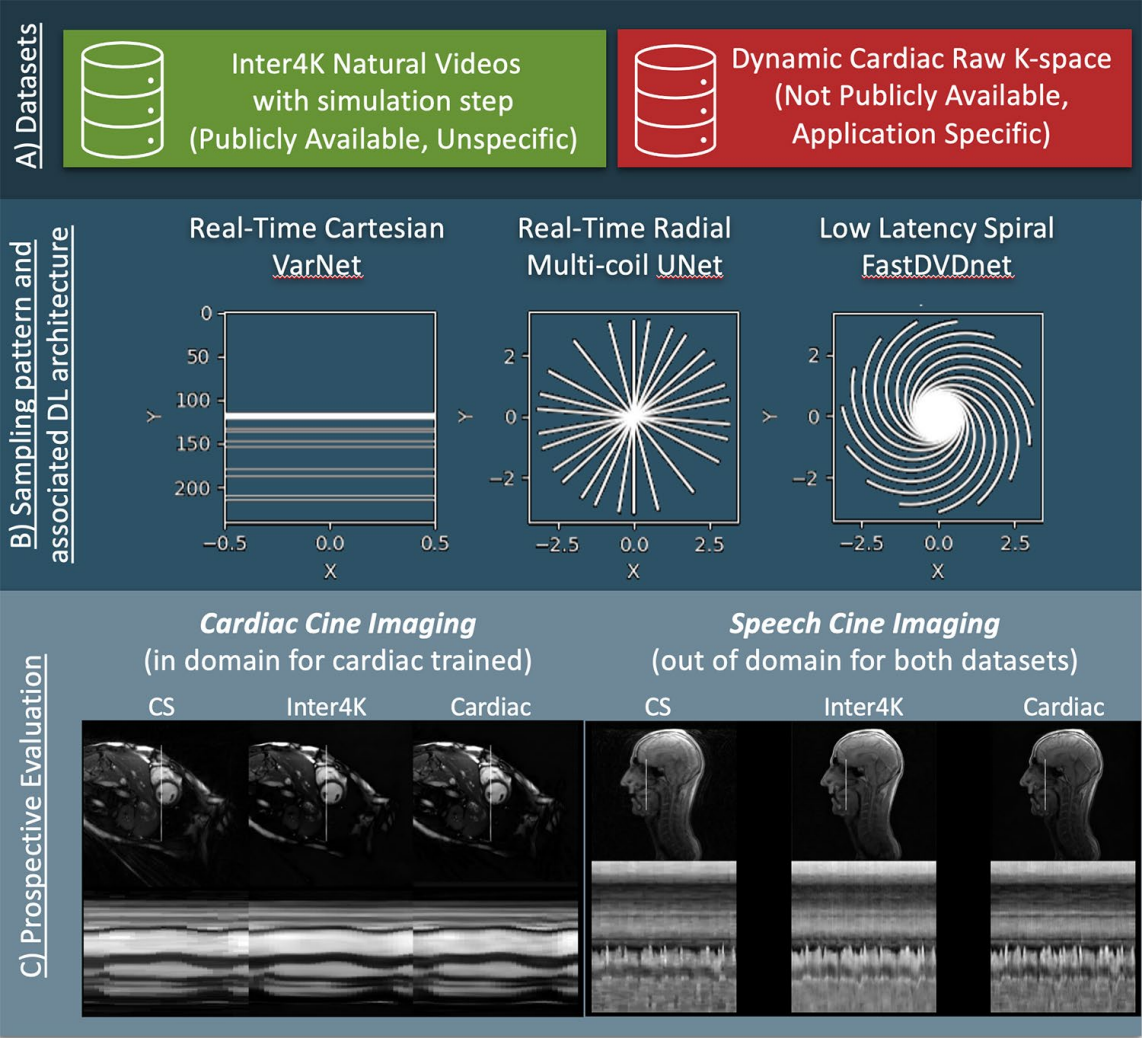
Experiment Overview. Training reconstructions from the natural video Dataset (Inter4K) was compared to training from a true cardiac MR dataset for three different sampling patterns and associated supervised DL reconstructions of real-time data: Cartesian acquisition with VarNet 2D + time reconstruction, radial acquisition with multi-coil 3D UNet reconstruction and spiral acquisition with FastDVDNet reconstruction. These methods were tested against Compressed Sensing (CS) reconstructions on two prospective applications: cardiac bSSFP in SAX and 4CH views (similar to cardiac training data) and Speech GRE cines (out of distribution for both cardiac and natural videos).

In this study, we aimed to demonstrate that training data created from natural videos (Inter4K) could be used to train a range of state-of-the-art iterative and deep artifact suppression DL approaches. These included iterative DL that is better suited to Cartesian acquisitions, and coil- combined and multi-coil image-based deep artefact suppression approaches that have previously been used for non-Cartesian data. Specifically these were: 1) an iterative unrolled VarNet^7,10^ with a 3D (2D + time) UNet regularizer for Cartesian real-time acquisitions, 2) a 3D (2D + time) multi-coil complex image-based UNet^6,11^ for tiny golden-angle radial real-time acquisitions, and 3) a magnitude-only, low-latency, image-based FastDVDNet^12^ network used for spiral real-time acquisitions (HyperSLICE^5^). Each DL method was trained separately on Inter4K data and cardiac MR data allowing comparison on both prospectively acquired real-time cardiac cine and speech cine data, along with CS reconstructions. An overview of the study is provided in Fig. 1.

### Training dataset preparation

#### Inter4K data

**Figure 2:**
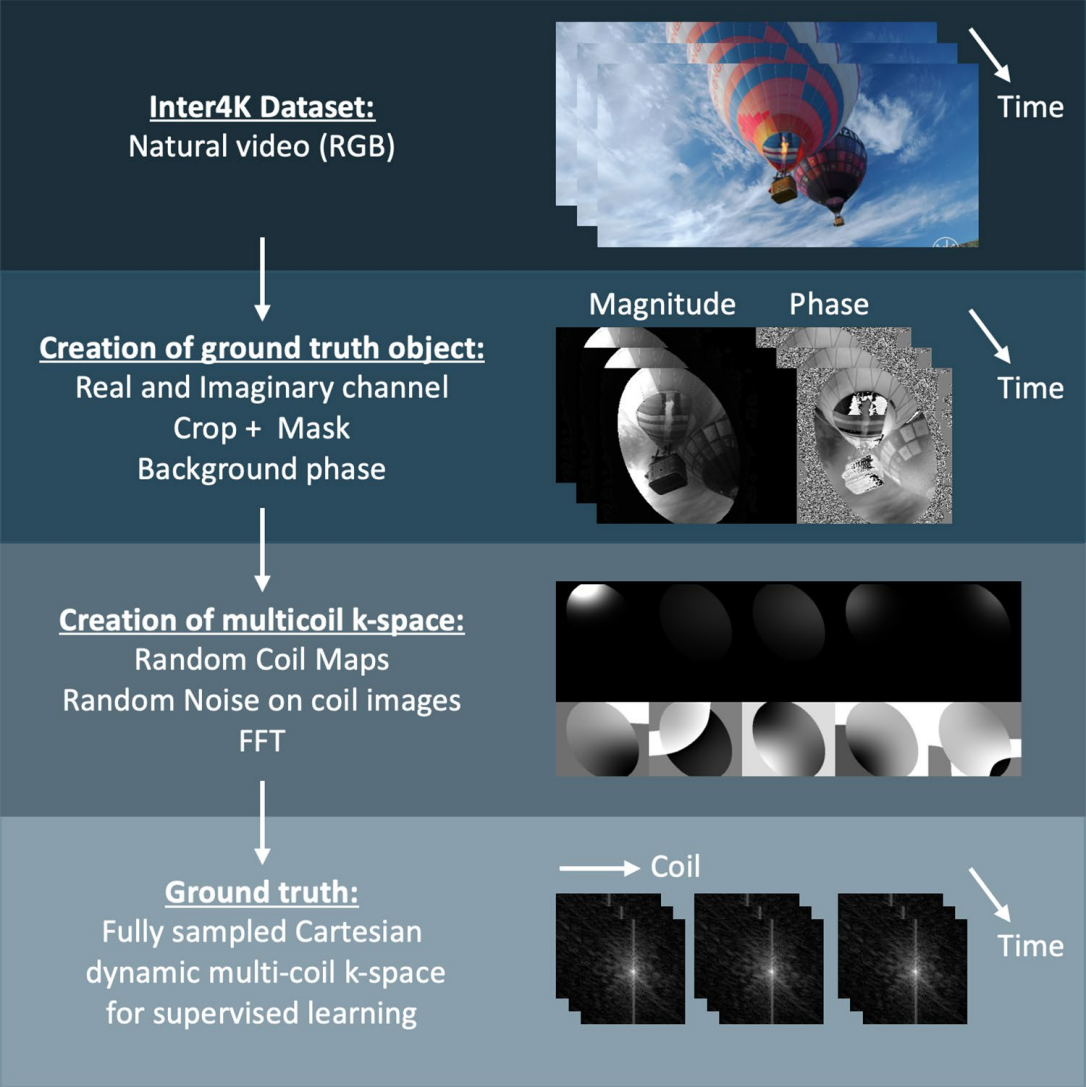
Pipeline for generating fully sampled Cartesian dynamic multi-coil k-space from natural videos. Step-by-step: **1** Creation of ground truth object: 2 of the 3 channel RGB video are randomly selected to create the real and imaginary parts and the phase between the two is scaled. Images are cropped and masked with an elliptical mask. Low frequency background phase is added. **2** Creation of ground truth multi-coil k-space: Random coil maps are generated and applied to the object, random noise is added on each coil image separately and finally a fast Fourier transform is applied to obtain the k-space data. **3** Finally, the data can be used as a regular input to any supervised reconstruction method.

Creation of synthetic ‘multi-coil’ k-space data from natural videos is summarized in Fig. 2. The Inter4K data set consists of one thousand 5-s RGB video clips at 4K resolution and 60 frames-per-second. To match the amount of cardiac MR training data we used 588 clips, from which we randomly selected 50 consecutive frames and downsampled to 488 × 868 pixels using 2D bilinear interpolation. We then randomly extracted two RGB channels to create the real and imaginary components of our synthetic image object, scaling the phase by 4 × to create a more realistic range and phase angles (factor chosen empirically). The complex image object was then cropped to the final size (depending on the acquisition being simulated) and masked with a randomly sized and rotated ellipse (long and short axis in range 1.0–1.4 × and 0.64–0.96 × the image width). Additional low frequency background phase was simulated by first creating a 6 × 6 random matrix, performing bicubic interpolation to the full image size, and finally added to the original image phase.

This synthetic image object was then converted to synthetic ‘multi-coil’ k-space data using the following simulated MR acquisition process. Firstly, 30 simulated random synthetic ‘coil maps’ were generated using 2D Gaussians with; i) random maximum intensity 0.1–1, ii) independent random standard deviation for x and y axes in the range 0.5–0.16 × the image size, iii) random center location (avoiding 1/5th of image center), and iv) random background phase offset. The final coil maps were normalized by the root-sum-of-squares of all the generated coil maps. Coil images were then obtained by multiplying each coil map with the image series and adding uniform random noise separately to each coil and timepoint image. The last step was to perform fast Fourier transformation to obtain synthetic fully sampled ‘multi-coil’ k-space data.

#### Cardiac MR data

All cardiac MR data were acquired in a single center on a 1.5T system (Aera, Siemens Healthineers, Erlangen, Germany) as previously described^5^. The training dataset consisted of 588 electrocardiogram-triggered breath-held Cartesian balanced steady state free precession (SSFP) CINE multicoil raw data (plus an additional 104 hold-out datasets for evaluation see below). The data set included seven different orientations: short axis, four chamber, three chamber, two chamber, right ventricular long axis, right ventricular outflow tract, and pulmonary artery. Data were collected in a diverse adult patient population (age: 58.7 ± 16.0 years, weight: 77.4 ± 18.6 kg, male/female: 56/36) referred for routine cardiovascular MR (including assessment for ischemia, cardiomyopathy, and pulmonary hypertension). The raw data were acquired with 2-times undersampling (with nominal matrix size of 224 × 272 and 44 autocalibration lines) and reconstructed with GRAPPA to recover fully sampled Cartesian multicoil k-space data.

#### Creation of paired training data

Processing steps for each architecture were the same for the cardiac MR data and natural videos and are described below. An example of synthetic undersampled data derived from natural videos for each investigated DL architecture/sampling pattern is shown in Supporting Information Video S1.

- *VarNet* The multi-coil k-space data (true and synthetic) was compressed to 10 virtual coils using Singular Value Decomposition. The compressed multi-coil k-space and then undersampled using a variable density Cartesian pattern with 17 lines per frame (~ R = 14). Each frame included the same 8 center lines, but 9 different random non-repeating lines sampled from the bottom 60% of k-space. This multi-frame, multi-coil undersampled k-space data acted as the input to the VarNet and the ground truth target was the coil combined (using coil sensitivities) multi-frame magnitude images from the fully sampled k-space. Prior to training the k-space data was normalized so that the coil combined magnitude image data was in the range 0–1.
- *3D UNet* The multi-coil k-space data was compressed as above and resampled onto 13 tiny golden angle (23.8°) radial spokes with continued increments between frames (~ R = 20). This radially undersampled multi-coil k-space data was then non-uniformally fast Fourier transformed (NUFFT) to produce the 2D + time multi-coil complex image input to the 3D UNet. The coil combined (root-sum-of-squares—RSS) 2D + time magnitude images derived from the fully sampled k-space data were used as the ground truth target. Prior to training the multi-coil complex image data was normalized so that the coil combined magnitude image data was in the range 0–1.
- *FastDVDNet* The multi-coil k-space data was compressed as above and resampled onto 15 uniform angle, variable density, spiral interleaves (average R ~ 6: R ~ 1.1 for the inner 15% of k-space and R ~ 15 for the outer 56% of k-space with a linear transition between). This undersampled data spiral multi-coil k-space data underwent NUFFT and RSS to create the 2D + time coil-combined magnitude input to FastDVDNet, with the ground truth target being the same as for the 3D UNet. Prior to training the coil-combined image data was normalized so that the magnitude was in the range 0–1.

### Network architectures and training

All networks were implemented using TensorFlow/Keras and leverage the open-source TensorFlowMRI which includes GPU-accelerated MR operations^13,14^. Training was the same for cardiac data and natural videos data, both of which were split 519/69 for training/validation/testing. Full details of the 3 network architectures are included in Supplementary Information Figs. 1, 2, 3. All networks were trained using a magnitude Structural Similarity Index Measure (SSIM) loss. An Adam optimizer with a learning rate of 10^–4^ was used to train all models for 100, 200 and 200 epochs for the VarNet, 3D Unet and FastDVDNet respectively. For training, 24 timeframes were used as input for the VarNet and 3D Unet, while the current and previous four frames were used for the FastDVDNet. Training was performed on a single NVIDIA A6000 GPU. The source code for our framework using natural videos (including creation of synthetic coils and synthetic multi-coil k-space data) and corresponding pre-trained networks are available at https://github.com/mrphys/Image_Reconstruction_Inter4k.git.

### Evaluation on simulated data

Evaluation using simulated data allowed comparison with the ground truth. Simulated undersampled data (for all 3 architectures/sampling patterns) were created from the 104 hold-out cardiac MR datasets pre-processed in the same way as the training data. Simulated undersampled data were then inputted into their respective DL models separately trained on either cardiac MR data or natural videos. In additions, the undersampled data underwent CS reconstruction with temporal Total Variation and a regularization factor of 5*10^–4^. The outputs were compared to ground truth data using Mean Squared Error (MSE), Peak Signal to Noise Ratio (PSNR) and SSIM.

### Evaluation on prospective data

Ten healthy subjects (30 ± 4 y.o., 73 ± 14 kg) underwent MR on a 1.5T system (same system as training data collected on). Approximately 10 s of real-time data were acquired in three separate scan planes: cardiac short axis (SAX), cardiac 4-chamber (4CH), and sagittal head (for speech cine imaging). Cardiac SAX and 4CH data were acquired using balanced steady-state free precession (bSSFP) during free-breathing without ECG-gating. Speech cine imaging was acquired with spoiled gradient echo (GRE) with subjects performing a simple number counting protocol (counting between 1 and 10 repeatedly). More information on the six different prospective real-time acquisitions can be found in Table 1. At inference, all undersampled prospective data (after normalization) were inputted into their respective DL models trained with either cardiac MR data or natural videos. As with the simulated data, all datasets were also reconstructed using CS with temporal Total Variation and a regularization factor of 5*10^–4^.

- *Qualitative image scoring evaluation* Qualitative comparison of the three reconstructions (DL trained using cardiac MR data, DL trained from natural videos, and CS) was performed by two clinical experts (V.M., D.K. with > 20 and 10yrs experience respectively). The three reconstructions were viewed simultaneously as a single movie clip separately for each scan plane and sampling pattern. The order of the reconstruction within the 3 × 1 frame was randomly shuffled per clip and scorers were blinded to the reconstruction type. The overall image quality of reconstructions in each clip were then subjectively ranked against each other from best to worse (1 = best, 2 = middle, 3 = worst, with possibility of tied scores).
- *Quantitative image scoring evaluation* As there was no ‘ground truth’ data for comparison, it was not possible to calculate MSE, PSNR and SSIM for prospective undersampled data. Thus, for each scan plane and sampling pattern the reconstructions were compared using estimated signal-to-noise ratio (SNR) and edge sharpness (ES). The estimated SNR was calculated as the mean signal intensity in the blood pool or tongue divided by standard deviation of pixels in a region outside the body. The ES was calculated as the maximum gradient along normalized intensity profile across the cardiac septum or tongue, and both the mean and standard deviation over time were reported. An example of SNR and ES measurements is provided in Supplementary Information Figure S4.

**Table 1:** Acquisition details for the six different prospective real-time acquisitions.

Trajectory	Sampling	Application	Type	Temporal resolution (ms)	Flip Angle (°)	TR (ms)	Spatial resolution (mm)	Field of view (mm)
Cartesian	17 lines	Cardiac	bSSFP	47	70	2.8	1.7 × 1.7	400
		Speech	GRE	58	15	3.4	1.7 × 1.7	400
Radial	13 spokes	Cardiac	bSSFP	38	70	3.0	1.6 × 1.6	400
		Speech	GRE	45	15	3.4	1.6 × 1.6	400
Spiral	15 variable density arms	Cardiac	bSSFP	55	70	3.7	1.7 × 1.7	400
		Speech	GRE	79	15	5.3	1.7 × 1.7	400

Cardiac and speech cine acquisitions had the same trajectories and were reconstructed using the same networks. *Cartesian*: 17 lines were acquired per timepoint including the 8 center lines and 9 randomly selected samples in bottom 60% of Fourier space. *Radial*: 13 radial spokes were sampled per timepoint with a constant tiny golden angle increment of 23.8°. *Spiral*: 15 variable density spiral arms were sampled per timepoint with a linear rotation between arms of 24° within a timepoint and an additional 30° increment between timepoints.

### Statistical analysis

As some of the distributions tested were non-normal (Shapiro–Wilk test), a Friedman Chi Square test with post-hoc Nemenyi analysis was used to assess any statistical differences (*p* < 0.05) between mean reported metrics.

## Results

### Training and inference

Training from natural videos and cardiac images took a similar amount of time, with VarNet, 3D UNet and FastDVDNet taking 32h, 18h and 1h30 to train respectively. Inference times were similar for both DL models (~ 10–100 × faster than CS, Supplementary Information Table S1).

### Evaluation on simulated data

Comparison of the ability of DL models trained with cardiac MR data and trained with natural videos to reconstruct simulated undersampled data are shown in Table 2 and Supplementary Information Fig. 4. The cardiac trained DL models outperformed those trained with natural videos in terms of MSE, PSNR and SSIM (*p* < 0.05). This was particularly true for the Cartesian undersampling (SSIM: 0.9 vs 0.83), but the difference was less marked for radial undersampling (SSIM: 0.93 vs. 0.88) and spiral undersampling (SSIM: 0.9 vs. 0.86). Both DL reconstructions outperformed CS reconstructions (Table 2).

**Table 2:** Comparison of different reconstructions (DL trained with natural video and cardiac MR data, as well as CS) for each sampling pattern for simulated cardiac images.

Acquisition	Reconstruction	MSE *10^4^	PSNR	SSIM
Cartesian	CS	15.72 ± 8.49	28.68 ± 2.39	0.8 ± 0.07
	Natural Videos	8.57 ± 4.67	31.18 ± 2.04	0.82 ± 0.06
	Cardiac	3.61 ± 2.3	35.16 ± 2.48	0.9 ± 0.05
Radial	CS	6.96 ± 5.58	32.63 ± 3.06	0.8 ± 0.07
	Natural Videos	3.67 ± 2.16	35.06 ± 2.45	0.88 ± 0.07
	Cardiac	2.01 ± 1.3	37.8 ± 2.63	0.93 ± 0.04
Spiral	CS	14.91 ± 14.49	29.38 ± 3.0	0.69 ± 0.08
	Natural Videos	6.51 ± 3.04	32.31 ± 1.95	0.86 ± 0.05
	Cardiac	4.15 ± 2.2	34.39 ± 2.26	0.9 ± 0.03

Mean and standard deviation for mean square error—MSE (in decibels -dB), peak signal–noise ratio—PSNR, and structural similarity index measure – SSIM. All results were statistically significantly different (*p* < 0.05).

### Evaluation on prospective data

**Figure 3:**
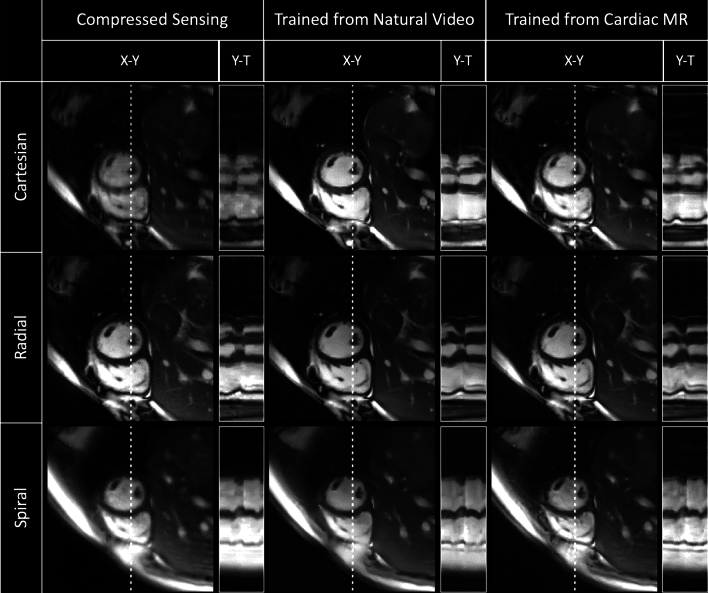
Qualitative comparison of a cardiac short axis cine dataset showing cropped image and Y–T profiles as indicated by white dotted line. From Top to Bottom: Real-time Cartesian, radial and spiral prospective acquisitions. From left to right: Compressed Sensing, natural video trained and Cardiac trained reconstructions. Deep learning architectures were VarNets, multi-coil 3D UNet, and low latency FastDVDNet for Cartesian, radial and spiral respectively. Corresponding video can be found in Supporting Information Video S2.

**Figure 4:**
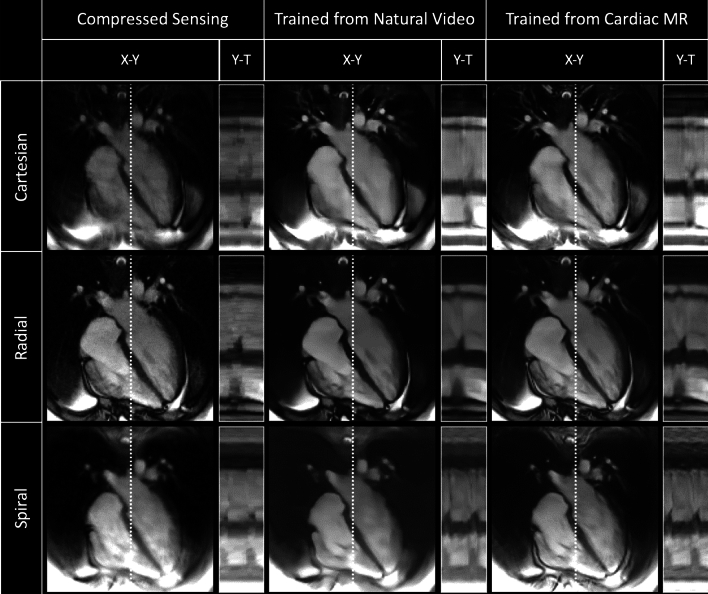
Qualitative comparison of a cardiac four chamber cine dataset showing cropped image and Y–T profiles as indicated by white dotted line. From Top to Bottom: Real-time Cartesian, radial and spiral prospective acquisitions. From left to right: Compressed Sensing, natural video trained and cardiac trained reconstructions. Deep learning architectures were VarNets, multi-coil 3D UNet, and low latency FastDVDNet for Cartesian, radial and spiral respectively. Corresponding video can be found in Supporting Information Video S3.

**Figure 5:**
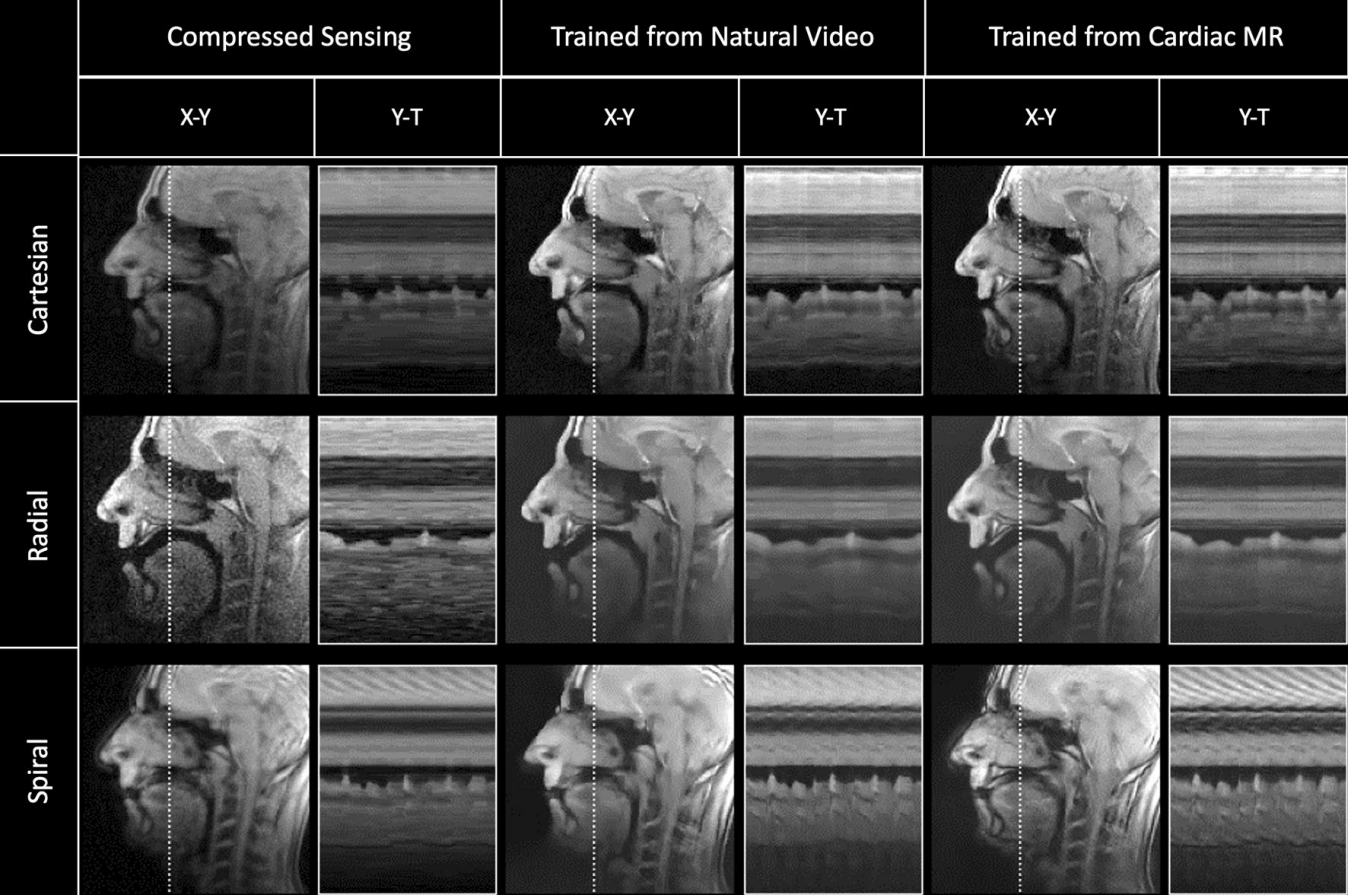
Qualitative comparison of a speech cine dataset showing cropped image and Y–T profiles as indicated by white dotted line. From Top to Bottom: Real-time Cartesian, radial and spiral prospective acquisitions. From left to right: Compressed Sensing, natural video trained and cardiac trained reconstructions. Deep learning architectures were VarNets, multi-coil 3D UNet, and low latency FastDVDNet for Cartesian, radial and spiral respectively. Corresponding video can be found in Supporting Information Video S4.

Comparison of the three reconstructions (DL trained from cardiac data, DL trained from natural videos, and CS) for each sampling pattern (Cartesian, radial, spiral) for representative SAX, 4CH and speech cine images are shown in Figs. 3, 4 and 5 respectively (with corresponding videos in Supplementary Information Videos S2, S3, S4).

Qualitative image scoring for each scan plane and sampling pattern are reported in Table 3 (cardiac cine data) and 3 (speech cine data). The DL reconstructions trained from natural videos ranked similarly to those trained from cardiac data (*p* > 0.19) except for Cartesian SAX and 4CH images where they ranked higher (*p* = 0.04). Both DL networks ranked significantly higher (*p* < 0.05) than CS, except spiral speech cines (although the same trend is observed). There no were statistically significant inter-rater differences (*p* > 0.42, Supplementary Information Table S2).

**Table 3:** Comparison of different reconstructions (DL trained with natural video and cardiac MR data, as well as CS) for each sampling pattern for prospective cardiac cine images (SAX N = 10, 4CH N = 10).

Cardiac cine	Reconstruction	SNR	Edge sharpness (ES)	Temporal STD ES	Subjective image quality ranking
Cartesian	CS	47.1 ± 27.4 *†	0.085 ± 0.021 †	0.016 ± 0.005	2.98 ± 0.16 *†
	Natural Videos	67.9 ± 19.0 *	0.106 ± 0.037	0.019 ± 0.006	1.2 ± 0.4 *
	Cardiac	164.8 ± 85.7	0.098 ± 0.024	0.018 ± 0.008	1.75 ± 0.49
Radial	CS	38.5 ± 18.7 *†	0.129 ± 0.032	0.017 ± 0.005	2.92 ± 0.35 *†
	Natural Videos	132.6 ± 71.9	0.127 ± 0.033	0.018 ± 0.007	1.3 ± 0.51
	Cardiac	207.0 ± 121.2	0.127 ± 0.029	0.017 ± 0.006	1.27 ± 0.45
Spiral	CS	66.2 ± 26.0 *	0.098 ± 0.028	0.015 ± 0.006 †	2.9 ± 0.3 *†
	Natural Videos	85.9 ± 37.2 *	0.104 ± 0.034	0.02 ± 0.007	1.55 ± 0.59
	Cardiac	108.7 ± 49.3	0.098 ± 0.024	0.017 ± 0.005	1.5 ± 0.59

Mean and standard deviation values for Signal–noise ratio—SNR (in decibels -dB), Edge Sharpness (mean over 1.5s), temporal standard deviation of edge sharpness through time (std over 1.5s) and subjective image quality ranking (between 1-best and 3-worst). *,† Statistically significantly different from cardiac and natural videos respectively (*p* < 0.05).

Across all scan planes and sampling patterns, there was no significant difference in SNR between DL reconstructions trained from cardiac MR data and natural videos (Tables 3 and 4), except for Cartesian speech cines (natural videos lower, *p* < 0.01) and spiral speech cines (natural videos higher, *p* = 0.02). Images reconstructed with CS either had similar or lower (*p* < 0.05) SNR than the DL reconstructions (Tables 3 and 4).

**Table 4:** Comparison of different reconstructions (DL trained with natural video and cardiac MR data, as well as CS) for each sampling pattern for prospective speech cine images (N = 10).

Speech cine	Reconstruction	SNR	Edge sharpness (ES)	temporal STD ES	Subjective image quality ranking
Cartesian	CS	14.0 ± 5.1 *	0.157 ± 0.023	0.046 ± 0.017	2.95 ± 0.22 *†
	Natural Videos	14.9 ± 3.7 *	0.169 ± 0.024	0.049 ± 0.018	1.15 ± 0.36
	Cardiac	26.7 ± 6.5	0.162 ± 0.031	0.046 ± 0.016	1.7 ± 0.46
Radial	CS	8.0 ± 1.9 *†	0.184 ± 0.032	0.045 ± 0.014	3.0 ± 0.0 *†
	Natural Videos	58.4 ± 15.3	0.183 ± 0.036	0.051 ± 0.018	1.15 ± 0.36
	Cardiac	76.4 ± 24.3	0.175 ± 0.029	0.045 ± 0.019	1.25 ± 0.43
Spiral	CS	41.8 ± 12.7 †	0.114 ± 0.014	0.033 ± 0.013	2.3 ± 0.84
	Natural Videos	61.0 ± 16.3	0.119 ± 0.018	0.034 ± 0.013	1.7 ± 0.78
	Cardiac	57.7 ± 26.2	0.117 ± 0.02	0.038 ± 0.018	2.1 ± 0.77

Mean and standard deviation values for Signal–noise ratio—SNR (in decibels -dB), Edge Sharpness (mean over 1.5s), temporal standard deviation of edge sharpness through time (std over 1.5s) and subjective image quality ranking (between 1-best and 3-worst). *,† Statistically significantly different from cardiac and natural videos respectively (*p* < 0.05).

For most comparisons, mean ES and the standard deviation of ES over time (STD-ES), between reconstructions were not significantly different (Tables 3 and 4, Figs. 3, 4, 5).

## Discussion

The main finding of this proof-of-concept study was that it was possible to train DL-based reconstructions for dynamic real-time MR data using open-source natural videos. We used the openly available Inter4K dataset from which we simulated synthetic MR data, enabling training of a range of state-of the-art complex multi-coil reconstructions (VarNet and 3D UNet) and magnitude only models (FastDVDnet). Importantly, we demonstrated that natural videos could be used to train iterative DL methods that are useful when data consistency is vital, as well as single pass methods that are useful for low latency applications.

For prospective data, we demonstrated that the subjective image quality of reconstructions from DL networks trained with natural videos and cardiac MR data were similar for both real-time cardiac (SAX and 4CH) and speech cine applications. In most cases this was also reflected in similar quantitative measures of edge sharpness and estimated SNR.

However, for the simulated undersampled cardiac MR test data, the natural videos DL reconstructions did have slightly lower image quality than cardiac DL reconstructions when compared to the ground truth. This is unsurprising as supervised DL tends to work better when test inputs (and desired outputs) have a similar distribution to the training data. Nevertheless, the fact that both DL models performed equally well on prospective data suggests that differences in simulation do not necessarily translate to differences during real-world inference.

The fact that natural videos trained DL models provide high quality reconstructions suggests that the underlying image object used for training doesn’t have to exactly replicate the test data. This is probably because the DL models that we investigated primarily ‘learn’ local image features and these are similar in both cardiac MR and natural video data. However, further study of the significance of the dissimilar image characteristics is required to improve our understanding of both the opportunities and limitations of learning from natural videos^15,16^. In addition, improving the quality and generalizability of these models could be explored through scaling up the number of training videos, or experimenting with noise and object phase addition. Another area that should be explored is the generation of synthetic multi-coil data, which was a novel aspect of this work. In our study, the simulation of the coil sensitivities was kept relatively straightforward for ease of computation and generalizability. The advantage of this approach was that no prior knowledge of coil geometries was required, which potentially enables easier application to other body parts/coil types. Nevertheless, future works that includes more realistic coil simulations via true MR acquisitions or generative DL^17^ could improve results.

Interestingly, we also demonstrated that cardiac MR based DL can successfully reconstruct speech cine images, demonstrating the potential of out of domain training with other types of MR data. These results suggest that cardiac MR trained DL models could be used for a wide range of dynamic non-cardiac applications and further strengthens the idea that training data doesn’t have to exactly reflect test data. Nevertheless, such models still require access patient data for training. On the hand, natural videos have higher underlying spatial and temporal resolution, as well as being open source, making data, results and pre-trained models easier to disseminate.

In this proof-of-concept study, we did not compare our approach with competing DL methods that use limited or no training data. The most commonly used method of mitigating a lack of training data is data augmentation and this approach has recently been further improved by creation of synthetic training data^17^. However, some training data is still required, which does these limit these methods. Newer approaches include zero-shot learning, deep image priors and geometry informed deep learning^18–20^ have shown great potential. However, these approaches represent a significantly different approach and are often limitated by long inference times. As our study was primarily aimed at demonstrating the potential of natural videos for training supervised methods, we did not perform comprehensive comparison with all alternative approaches. Another limitation of this study is that we did not compare cardiac volumes from the different reconstructions. This was beyond the scope of this proof-of-concept study but is required to truly demonstrate that natural videos provide robust clinically useful reconstructions. A final limitation of this study was that we did not directly compare the different sampling strategies. This was not performed because our aim was not to investigate optimum k-space sampling, rather we attempted to show that natural videos could be used across a broad range of applications and acquisitions.

## Conclusion

A proof-of-concept pipeline to learn dynamic MR image reconstruction from publicly available natural videos was applied to a variety of trajectories with different network architectures showing no significant differences or better subjective image quality compared to similar reconstructions trained on true dynamic cardiac MR data.

### Supplementary Information

Supplementary Information 1.

Supplementary Video 1.

Supplementary Video 2.

Supplementary Video 3.

Supplementary Video 4.

## Data Availability

The source code for training and testing our framework using natural videos and corresponding pre-trained networks for dynamic MRI reconstruction are provided online (https://github.com/mrphys/Image_Reconstruction_Inter4k.git).
