# Supplementary material for: Training deep learning based dynamic MR image reconstruction using open-source natural videos
Source: Sci Rep. 2024 May 23;14:11774. doi: 10.1038/s41598-024-62294-7 (PMC11116488; doi:10.1038/s41598-024-62294-7)
Supplement: Supplementary file 1 — Supplementary Information 1. [file 41598_2024_62294_MOESM1_ESM.pdf]

Supplemental Material

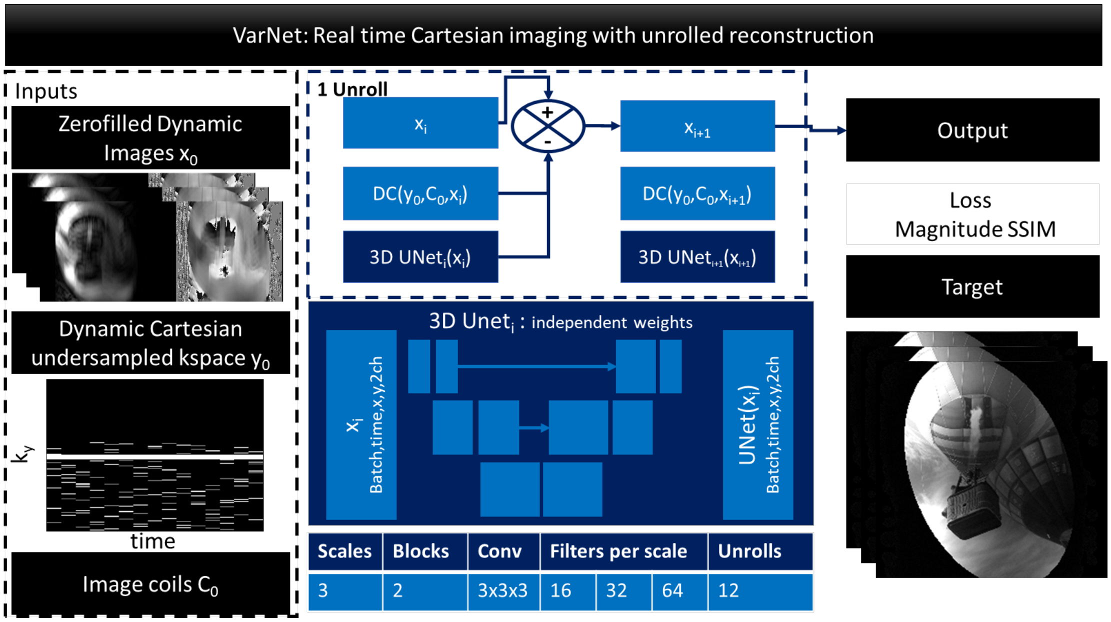

Scales	Blocks	Conv	Filters per scale			Unrolls
3	2	3x3x3	16	32	64	12

Supplementary Information Figure S1. Summarized method for the Cartesian acquisition with VarNet reconstruction network. The inputs consist of 24 zero-filled coil combined consecutive images as initialization x_0, corresponding complex multi-coil k-space y_0 with 17 lines per timepoint (including 8 center lines + 9 randomly non-repeating lines sampled in bottom 60% of k-space), and coil sensitivities estimated from the time combined data. The VarNet architecture was extended to 2D+time by applying a 3D UNet for regularization. Parameters are included in the bottom table. The network was trained using an ADAM optimizer for 100 epochs using a Magnitude SSIM loss.

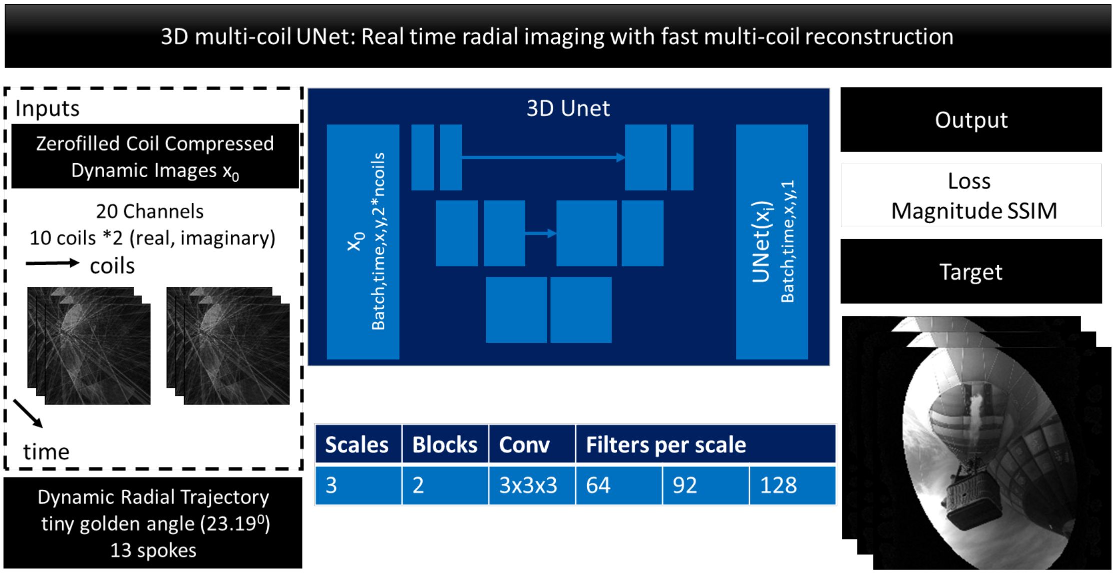

Supplementary Information Figure S2. Summarized method for the Radial acquisition with multi-coil 3D UNet reconstruction. The input consists of 24 gridded complex consecutive images coil compressed to 10 coils. Each frame was acquired using 13 spokes incremented by the tiny golden angle in k-space (23.19º). UNet parameters are included in the bottom table. The network was trained using an ADAM optimizer for 200 epochs using a Magnitude SSIM loss.

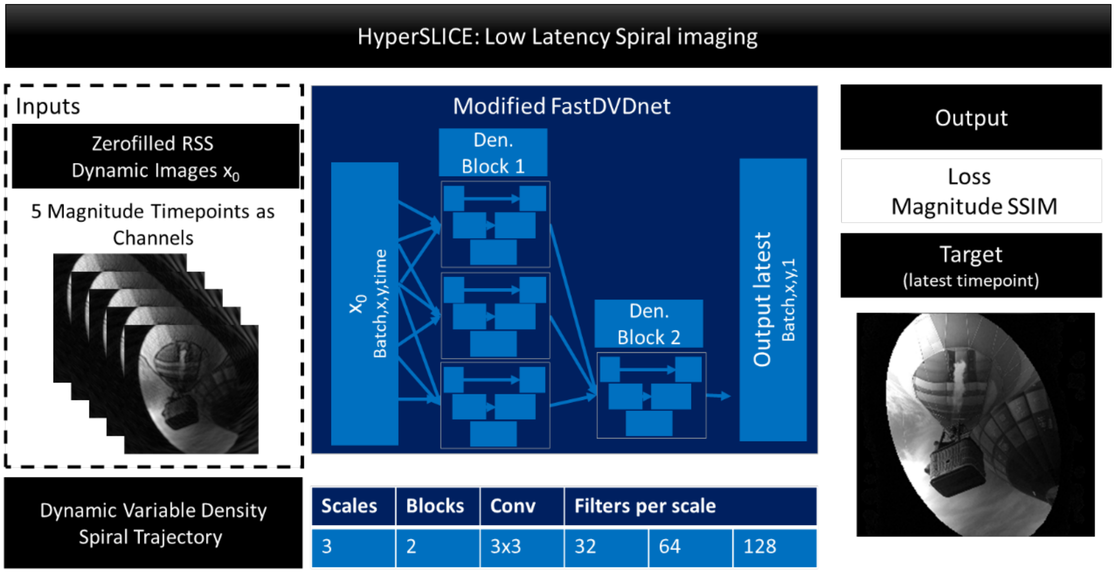

Supplementary Information Figure S3. Summarized method for the spiral acquisition with low latency FastDVnet reconstruction. The input consists of the five latest gridded root-sum-of-squares magnitude images. Each frame was acquired using a variable density spiral trajectory in k-space. Denoising block parameters are included in the bottom table. The network was trained to reconstruct the latest timepoint using an ADAM optimizer for 200 epochs using a Magnitude SSIM loss. More details on the acquisition and reconstruction can be found in (11).

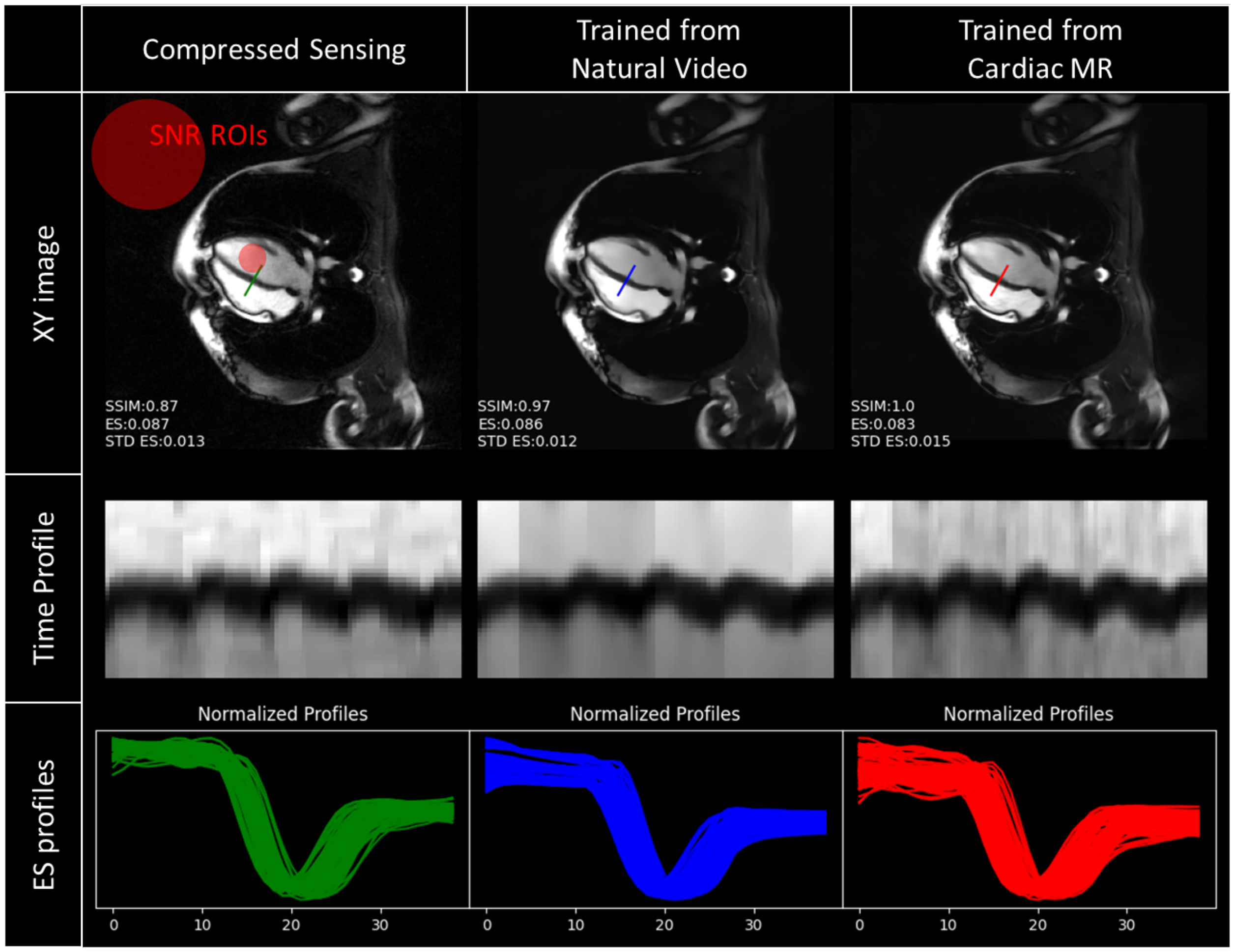

Supplementary Information Figure S4. Example of quantitative measurements performed in one radial prospective 4chamber dataset for the three reconstructions considered: Compressed Sensing with temporal TV regularization, 3D UNet trained with cardiac data and 3D UNet trained with natural videos. Representative examples of measurements of SNR (ROIs) and edge sharpness mean (ES) and temporal standard deviation (STD ES) using the maximum gradient of the normalized profiles across the septum for each timepoint.

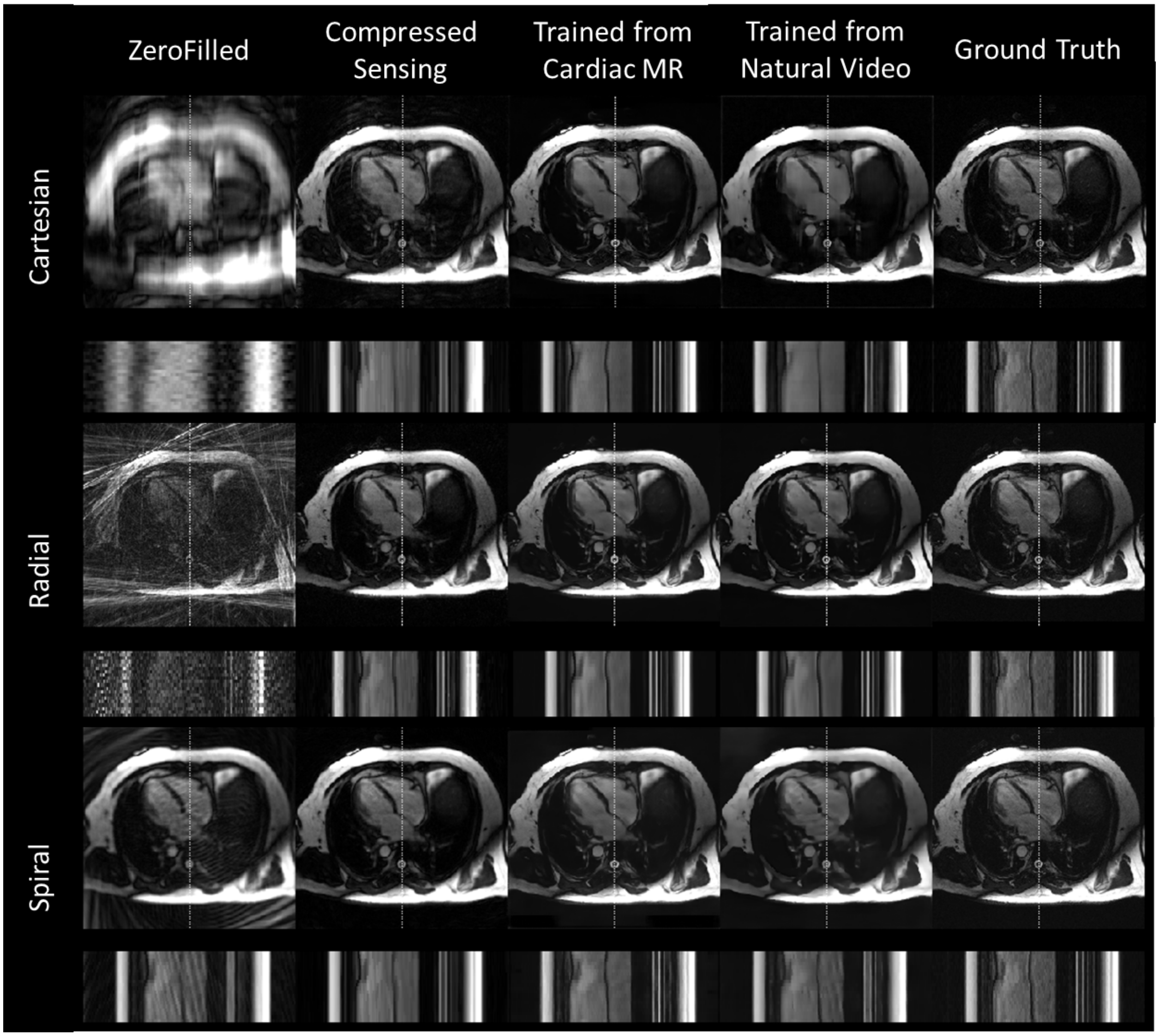

Supplementary Information Figure S5. Qualitative Results in one test set comparing Zero-filled, Compressed Sensing, cardiac trained and natural video trained reconstructions to the Ground Truth for the three methods considered (Cartesian VarNet, Radial 3D UNet, Spiral FastDVDNet).

Acquisition	Reconstruction	Inference time/batch	Inference time/frame
**Cartesian**	**CS Temporal TV**	8.2s	341ms
	**VarNet Natural Videos**	0.9s	38ms
	**VarNet Cardiac**	0.9s	38ms
**Radial**	**CS Temporal TV**	10.04s	430ms
	**3D UNet Natural Videos**	0.11s	5ms
	**3D UNet Cardiac**	0.11s	5ms
**Spiral**	**CS Temporal TV**	33.4s	1395ms
	**FastDVDnet Inter4k**	-	22ms
	**FastDVDnet Cardiac**	-	22ms

Supplementary information Table S1. Inference Times details for different reconstructions. The VarNet and Unet and CS reconstructions operated on 24 frames of data in a batch and therefore, Inference time per batch is reported. We also calculated inference time per frame. FastDVDnet was a frame-by-frame reconstruction so only inference time per frame is reported.

	Rater 1	Rater 2	Wilcoxon Signed rank test p-value.
**CS**	2.867	2.878	0.914
**Cardiac**	1.311	1.378	0.424
**Natural Videos**	1.544	1.589	0.498

Supplementary information Table S2. Inter-rater subjective image quality scores averaged across all sampling patterns and applications (e.g. cardiac and speech cine imaging). Wilcoxon signed rank test was used to compare ratings for the different methods and showed no statistically significant difference between raters.

Supplementary Information Video S1. Overview of Inter4K video and reconstructions from a test sample. Top: Cropped RGB, undersampled Cartesian zero-filled reconstruction, undersampled radial zero-filled reconstruction, undersampled spiral zero-filled reconstruction. Bottom: Target magnitude image, VarNet reconstruction, multi-coil 3D Unet reconstruction, FastDVDnet reconstruction.

Supplementary Information Video S2. Video comparison of a short axis cardiac dataset. From Top to Bottom: Real-time Cartesian, radial and spiral prospective acquisitions. From left to right: Compressed Sensing, natural video trained and cardiac trained reconstructions. Machine learning reconstructions were VarNets, multi-coil 3D UNet, and low latency FastDVDNet for Cartesian, radial and spiral respectively.

Supplementary Information Video S3. Video comparison of a four chambers cardiac dataset. From Top to Bottom: Real-time Cartesian, radial and spiral prospective acquisitions. From left to right: Compressed Sensing, natural video trained and cardiac trained reconstructions. Machine learning reconstructions were VarNets, multi-coil 3D UNet, and low latency FastDVDNet for Cartesian, radial and spiral respectively.

Supplementary Information Video S4. Video comparison of a speech dataset. From Top to Bottom: Real-time Cartesian, radial and spiral prospective acquisitions. From left to right: Compressed Sensing, natural video trained and cardiac trained reconstructions. Machine learning reconstructions were VarNets, multi-coil 3D UNet, and low latency FastDVDNet for Cartesian, radial and spiral respectively.
